# Demonstration of Cytotoxicity against Wasps by Pierisin-1: A Possible Defense Factor in the Cabbage White Butterfly

**DOI:** 10.1371/journal.pone.0060539

**Published:** 2013-04-23

**Authors:** Azusa Takahashi-Nakaguchi, Yasuko Matsumoto, Masafumi Yamamoto, Kikuo Iwabuchi, Yukari Totsuka, Takashi Sugimura, Keiji Wakabayashi

**Affiliations:** 1 Cancer Prevention Basic Research Project, National Cancer Center Research Institute, Tokyo, Japan; 2 Medical Mycology Research Center, Chiba University, Chiba, Japan; 3 Drug Discovery Imaging Platform Unit RIKEN Center for Molecular Imaging Science, Hyogo, Japan; 4 Faculty of Agriculture, Tokyo University of Agriculture and Technology, Tokyo, Japan; 5 Graduate Division of Nutritional and Environmental Sciences, University of Shizuoka, Shizuoka, Japan; University of South Florida College of Medicine, United States of America

## Abstract

The cabbage white butterfly, *Pieris rapae*, produces pierisin-1, a protein inducing apoptosis of mammalian cells. In the present study, the biological activity of pierisin-1 as a protective agent against parasitic wasps for *P. rapae* was examined. Pierisin-1 caused detrimental effects on eggs and larvae of non-habitual parasitoids for *P. rapae*, *Glyptapanteles pallipes*, *Cotesia kariyai* and *Cotesia plutellae* at 1–100 µg/ml, levels essentially equivalent to those found in *P. rapae* larvae. In contrast, eggs and larvae of the natural parasitoid of *P. rapae*, *Cotesia glomerata* proved resistant to the toxicity of pierisin-1 through inhibition of pierisin-1 penetration of the surface layer. The expression level of pierisin-1 mRNA in the larvae of *P. rapae* was increased by parasitization by *C. plutellae*, whereas it was decreased by *C. glomerata*. In addition, *C. plutellae* was associated with elevation of activated pierisin-1 in the hemolymph. From these observations, it is suggested that pierisin-1 could contribute as a defense factor against parasitization by some type of wasps in *P. rapae.*

## Introduction

Pierisin-1 is a cytotoxic protein found in the cabbage white butterfly, *Pieris rapae* (Lepidoptera: Pieridae) [1], [2]. Incorporated into mammalian cells expressing glycosphingolipid receptors such as globotriaosylceramide (Gb3) and globotetraosylceramide (Gb4) [3], pierisin-1 catalyzes ADP-ribosylation of guanine residues of DNA, then induces apoptosis [4]. Pierisin-1 is a 98-kDa protein comprising 850 amino acids featuring an N-terminal region (27-kDa) catalyzing ADP-ribosylation and a C-terminal region (71-kDa) with receptor-binding ability. We have reported that pierisin-like proteins, showing cytotoxicity and DNA ADP-ribosylating activity, are exclusively present in some specific genus of Pieridae (Pierina, Aporiina and Appiadia) [5], [6], [7]. The mosquitocidal toxin (MTX) from *Bacillus sphaericus* SSII-1 exhibits a sequence similarity to pierisin-1 throughout the coding region [1], [8]. Therefore, pierisin genes might have been transferred from insect pathogenic bacteria to the last common ancestor of the three genus of Pieridae butterfly. The amounts of pierisin-1 protein are increased around 100 times from the first-instar to fifth-instar larvae and then gradually decreased by over 90% during the pupal stage. Pierisin-1 is mainly located in fat bodies of fifth- instar larvae and early-phase pupae [9]. Based on the above data, it is suggested that pierisin-1 may play important roles in induction of apoptosis to remove larval cells in the pupation of *P. rapae*. Another possibility is that the strong cytotoxicity of pierisin-1 might be effective as a protective agent against microbes and/or parasitoids. However, the actual biological significance of pierisin-1 in *Pieris* butterflies has yet to be fully elucidated.

Insects rely on humoral and cellular defense reactions against intruding parasites and pathogens. Humoral immune responses mainly involve the synthesis of antimicrobial proteins [10], [11] and melanization [12], [13]. On the other hand, cellular immune responses include phagocytosis, nodule formation and encapsulation [14], [15], [16], [17]. It is well documented that encapsulation and melanization are major defense responses against eggs and larvae of parasitoid wasps [18], [19], [20], [21]. Quinones and reactive oxygen intermediates generated during the melanization process are presumed to be responsible for parasitoid death [11], [22], and several viruses and symbiotic bacteria produce toxic factors to eliminate competitive parasitic wasps [23], [24], [25], [26], [27], [28], [29]. However, no components in insect hemolymph directly causing damage to parasitoid cells have hitherto been identified.

The endoparasitic wasp, *Cotesia glomerata* (Hymenoptera: Braconidae) is a larval parasitoid of the cabbage white butterfly. To understand the role of pierisin-1 in the host defense responses, the present study was designed to clarify its direct toxicity of pierisin-1 against *C. glomerata* and other Braconid wasps *in vitro.* We found that pierisin-1 was able to cause damage to eggs and larvae of non-habitual parasitic wasps, *Glyptapanteles pallipes*, *Cotesia kariyai* and *Cotesia plutellae*, whereas the habitual parasitic wasp *C. glomerata* could evade damage. Since pierisin-1 showed cytotoxicity to cells of *C. glomerata* after removal of the eggshell or surface layer of the larval caudal vesicle, the results suggested that pierisin-1 permeability might be reduced. In addition, we also reported here fluctuation of pierisin-1 mRNA expression and pierisin-1 protein states in hemolymph after parasitization. Based on these results, the biological significance of pierisin-1, as a defense factor in Pieridae insects is discussed.

## Materials and Methods

### Insects

Adult females of *P. rapae* (Lepidoptera: Pieridae) were collected from fields in Tokyo and Tochigi Prefectures, Japan and *C. glomerata* (Hymenoptera: Braconidae) were obtained from parasitized host larvae. *Cotesia plutellae* (Hymenoptera: Braconidae) and its host *Plutella xylostella* (Lepidoptera: Plutellidae), and *Cotesia kariyai* (Hymenoptera: Braconidae) and its host *Pseudaletia separata* (Lepidoptera: Noctuidae), were kindly provided by Dr. Masayoshi Uefune (Kyoto University) and Dr. Madoka Nakai (Tokyo University of Agriculture and Technology), respectively. *Glyptapanteles pallipes* (Hymenoptera: Braconidae) and its host *Ctenoplusia agnate* (Lepidoptera: Noctuidae) were obtained from Dr. Daisuke Uka (Tokyo University of Agriculture and Technology). Hatched larvae of *P. rapae* and *P. xylostella* were reared on kale leaves (23 ± 2°C; 16 h photophase). *P. separata* and *C. agnate* were reared on artificial diets (Insecta). Adults of parasitoids were separately maintained in glass tubes containing honey as food, and kept in a climate-controlled room (15 ± 1°C, 24 h darkness) until use. No specific permits were required for the described field studies.

### Pierisin-1 toxicity assay against parasitic wasps

For *in vitro* toxicity bioassays, host eggs which had completed segment formation and first instar larvae (2 - and 4 -days-old) were prepared as follows. Parasitized host larvae were surface-sterilized in 70% ethanol solution, and then dissected in TC-100 medium (Kohjinbio, Japan) with 10% FBS. Parasitoid eggs and larvae collected from the host larvae were washed twice in the medium and two eggs or larvae were each transferred into 60 µl of TC-100 medium in 96 well plates.

Parasitoid eggs and larvae were cultured in medium containing pierisin-1 at the final concentrations of 1, 10 and 100 µg/ml for 2 and 7 days, respectively, and then microscopically observed. As a negative control, BSA was added to medium instead of pierisin-1. Values represent the average of 3–5 independent assays. Wasps were first observed morphologically using a phase-contrast microscope and then ultrastructurally. Wasps were prefixed with 2% (w/v) glutaraldehyde containing 0.1 M phosphate buffer at 4°C for 10 hr, followed by 1% osmium tetraoxide in phosphate buffer for an additional 1 hr. After dehydration, tissues were embedded in epoxy resin. Ultrathin sections were stained with uranyl acetate and lead citrate, and were visualized under an H-7500 transmission electron microscope (Hitachi,Japan).

For cytotoxicity assays of cells within *C. glomerata* larvae, the surface layer of the caudal vesicle was removed by pipetting with a glass capillary, and then the inner cells were incubated with HiLyte Flour-555 (Dojindo Molecular Technologies, Inc., Japan) -labeled pierisin-1 at 100 µg/ml.

### Transmigration assay of pierisin-1

Three developmental stages of wasps (early stage eggs: cellular blastoderm stage, late stage eggs: completed segment formation but not with cuticle, and second instar larvae) were used for culturing in a medium containing pierisin-1 or BSA labeled with HiLyte Flour-555 at a final concentration of 10 µg/ml. Cultured wasps were labeled with STYO-16 Live-Cell Nucleic Acid Stain (Molecular Probes,USA) and analyzed with a Leica TCS laser scanning confocal microscope after 24 hr.

### Preparation of RNA and protein

Third instar larvae of *P. rapae* at day 0 (3–4 mg body weight) were parasitized by *C. glomerata* or *C. plutellae.* Total RNA from the whole bodies of these host larvae was isolated using an RNeasy® Mini Kit (Qiagen). Plasma proteins (1 µl) of parasitized third instar Day 0 (L3D0) larvae were separated from hemocytes by centrifugation at 390 × g for 5 min at 4°C with 19 µl of anti-coagulant buffer (98 mM NaOH, 186 mM NaCl, 1.7 mM EDTA, 41 mM citric acid, pH 4.5).

### Quantification of pierisin-1 mRNA by quantitative RT-PCR

Synthesis of cDNA from total RNA was carried out with ReverTra Ace and random primers (Toyobo, Japan) then real-time PCR was carried out in 96 -well plates with a 20 µl reaction volume containing 10 µl 2×SYBR® Green Realtime PCR Master Mix (Toyobo), 2 µM of each forward and reverse primer, and 1 µg of cDNA. The samples were subjected to denaturation at 95°C for 1 minute, followed by 40 cycles of amplification (95°C for 20 s, 60°C for 20 s and 72 °C for 20 s) using DNA Engine Opticon2 (MJ Research, USA). Expression of pierisin-1 mRNA was normalized by 18S ribosomal RNA as an internal control. Values represent the average of 3–5 independent assays, and were statistically analyzed using the Student's t-test. The primer combinations were as follows: 5′- CATAACGGACGCATTCAAAG -3′ (forward primer) and 5′- GGTTTTGGGTTATTAGGCTC -3′ (reverse primer) for pierisin-1 gene, 5′- ACAATTGGAGGGCAAGTCTG -3′ (forward primer) and 5′- CACCGCGATAGGATTTTGAT -3′ (reverse primer) for 18S ribosomal RNA gene.

### Quantification of pierisin-1 protein levels by western blotting

Proteins of the samples including whole body or tissue of host larvae were separated using SDS-PAGE, and transferred on to polyvinylidene difluoride membranes (Immobilon-P; Millipore Corporation, Bedford, USA). Anti-pierisin-1 antibodies [30] that bind N-terminal region of pierisin-1 were diluted 1∶10^4^ in blocking solution (Block Ace; Dai-nippon Pharmaceutical Corp., Japan) in Tris-buffered saline containing 0.1% Tween-20, and secondary alkaline phosphatase antibodies conjugated to goat anti-rabbit IgG were diluted 1∶10^4^ in the same blocking buffer, followed by addition of ECF substrate (ECF Western Blotting Reagent Pack, Rabbit, GE Healthcare). Images were digitalized using a LAS-3000 imaging system (Fujifilm, Japan) and analyzed by Multi Gauge Ver. 2.0 (Fujifilm, Japan).

## Results

### Toxicity of pierisin-1 against in vitro cultured parasitoid eggs

**Figure 1:**
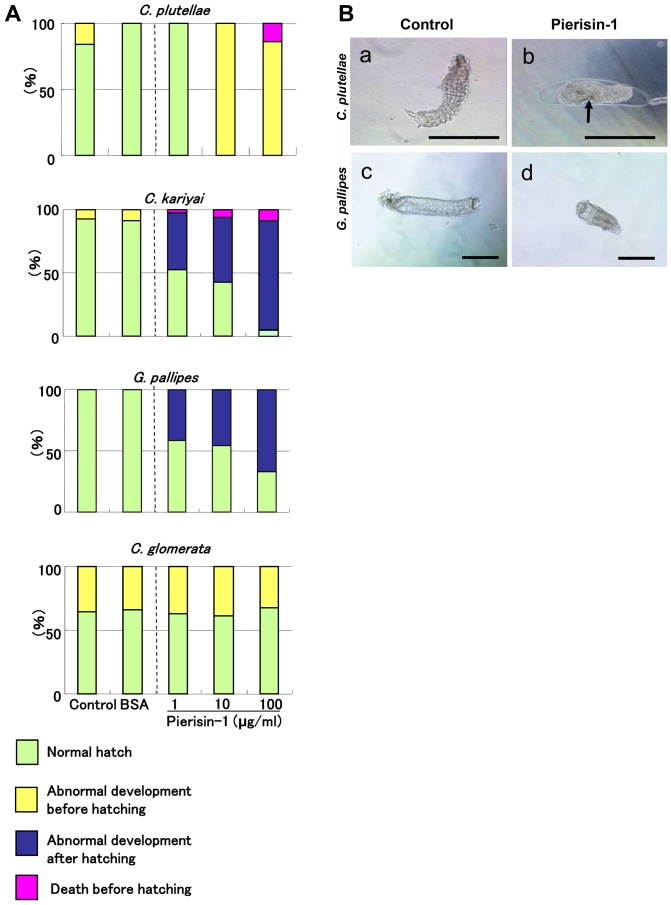
Effects of pierisin-1 on parasitoid eggs. Segment formation completed parasitoid eggs were cultured for 2 days in media with or without pierisin-1. A: Rates of damaged eggs of parasitic wasps. Green column, normal hatch; yellow column, abnormal development before hatching; blue column, abnormal development after hatching; red column, death before hatching. Most *C. kariyai* and *G. pallipes* hatched in medium containing pierisin-1, but the larvae developed abnormally. Around 70% of *C. glomerata* hatched and developed normally. Some of them could not hatch but actively moved in the eggshell under these culture conditions. Therefore, we counted these wasps as “Abnormal development before hatching”. The numbers of eggs used for each treatment were 9 –13 for *C. plutellae*, 22 – 34 for *C. kariyai*, 10 – 26 for *G. pallipes*, 31 – 45 for *C. glomerata*. B: Phase contrast microscope images of cultured wasps. Embryo development of most *C. plutellae* was suppressed and cell death (arrow) was also observed with pierisin-1 treatment (a, b). *G. pallipes* larval length was shortened compared with the controls (c, d). Wasp eggs (n = 8 for each wasps) were treated with pierisin-1 at a final concentration of 10 µg/ml in the medium, and representative microscope images are shown. The anterior is on the left. Horizontal bars represent 200 µm.

To investigate the toxicity of pierisin-1, we prepared four species of parasitoids belonging to Braconidae. Three of them, *C. glomerata, C. plutellae* and *C. kariyai* belong to the same *Cotesia* genus. *C. glomerata* naturally oviposits their eggs into *P. rapae. C. plutellae* occasionally mis-oviposits their eggs into *P. rapae* larvae but these eggs cannot complete normal development in *P. rapae.* The other, *G. pallipes* also belongs to Braconidae but not the *Cotesia* genus. *C. kariyai* and *G. pallipes* are not natural parasitoids of *P. rapae*. These four kinds of wasp eggs after complete segment formation, therefore at a late stage, were cultured *in vitro*, and incubated with 1, 10 and 100 µg/ml pierisin-1, because the concentrations of the toxin in the larval hemolymph of *P. rapae* vary from 5–50 µg/ml in first- to fifth-instar larvae. Pierisin-1 caused detrimental effects on non-habitual parasitoids, *C. plutellae*, *C. kariyai* and *G. pallipes* (Fig. 1A). *C. plutellae* eggs could not hatch and embryonic cell death was observed at 10 and 100 µg/ml pierisin-1. Most eggs of *C. kariyai* and *G. pallipes* were able to hatch in the presence of pierisin-1 at 1–100 µg/ml. However, the larvae showed abnormal morphology, such as shortening, and midgut and body dilatation. These morphological abnormalities might occur through defects in osmotic pressure caused by cell death in the gut (Fig. 1B).

Effects of pierisin-1 at 1–100 µg/ml on eggs of *C. glomerata* were similar to those of control and BSA treatment (Fig.1A). About 30% of *C. glomerata* eggs failed to hatch in the pierisin-1-containing medium, while all new larvae lived within egg shells. BSA treatment did not adversely affect any eggs of these four parasitoid species (Fig. 1B).

### Toxicity of pierisin-1 to parasitoid larvae

**Figure 2:**
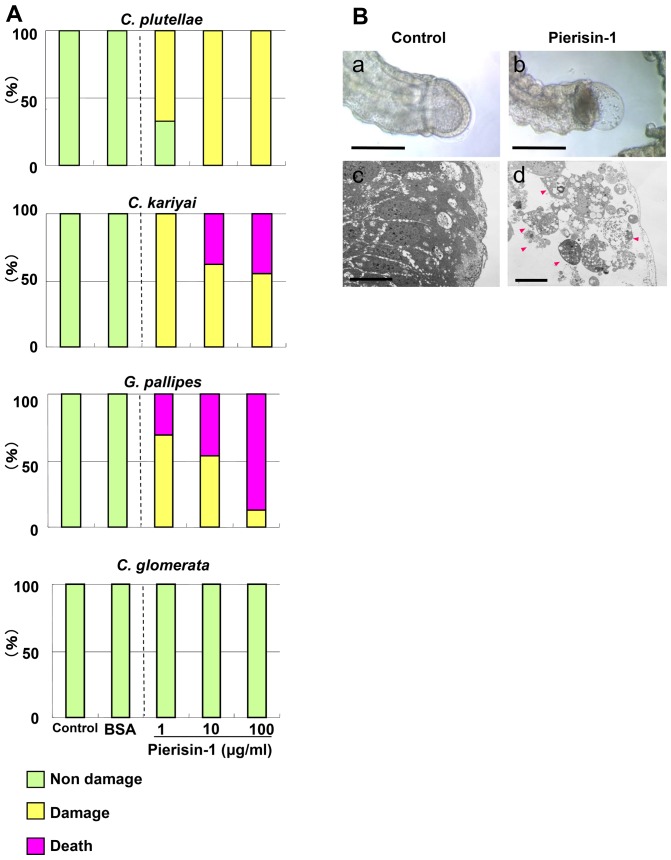
Effects of pierisin-1 on parasitoid larvae. First instar larvae were cultured for 7 days in media with or without pierisin-1. A: Rates of damaged larvae of parasitic wasps. Green column, non-damage; yellow column, damaged; red column, death. *C. glomerata* larvae developed normally even in the presence of pierisin-1. Pierisin-1 treatment caused damage in larval bodies of non-habitual wasps. The numbers of larvae used for each treatment were 9 – 10 for *C. plutellae*, 11 – 16 for *C. kariyai*, 9 – 16 for *G. pallipes*, 11 – 25 for *C. glomerata.* B: Morphological analysis of cultured *C. kariyai.* a and b: Phase contrast microscope images of cultured *C. kariyai* larva with or without pierisin-1, respectively. c and d:. Transmission electron microscope (TEM) images of cross sections of their caudal vesicles. Apoptosis was observed in caudal vesicles on pierisin-1 treatment (arrowheads). Wasp larvae (n = 16) were treated with pierisin-1 at 10 µg/ml in the medium, and representative microscope images are shown. The anterior is on the left. Horizontal bars for phase contrast microscope represent 100 µm. Bars for TEM represent 1 µm.

First instar larvae of parasitoids were cultured and pierisin-1 toxicity to these larvae was examined. All larvae actively moved in the medium and seemed not to be adversely affected by culture with or without BSA even after 7 days. *C. plutellae*, *C. kariyai* and *G. pallipes* larvae were highly sensitive to pierisin-1, and most of them were damaged by pierisin-1 treatment even at a dose of 1 µg/ml (Fig. 2A). The mortality rates of *C. kariyai* and *G. pallipes* were clearly increased at doses of 10 and 100 µg/ml. As shown in Fig.2B, apoptosis - like damage was observed in caudal vesicle cells of *C. kariyai* and *G. pallipes* larvae. In contrast, *C. glomerata* larvae were resistant to pierisin-1 at doses of 1, 10 and 100 µg/ml, and no histological damage was observed.

### Penetration of pierisin-1 into parasitoid cells

**Figure 3:**
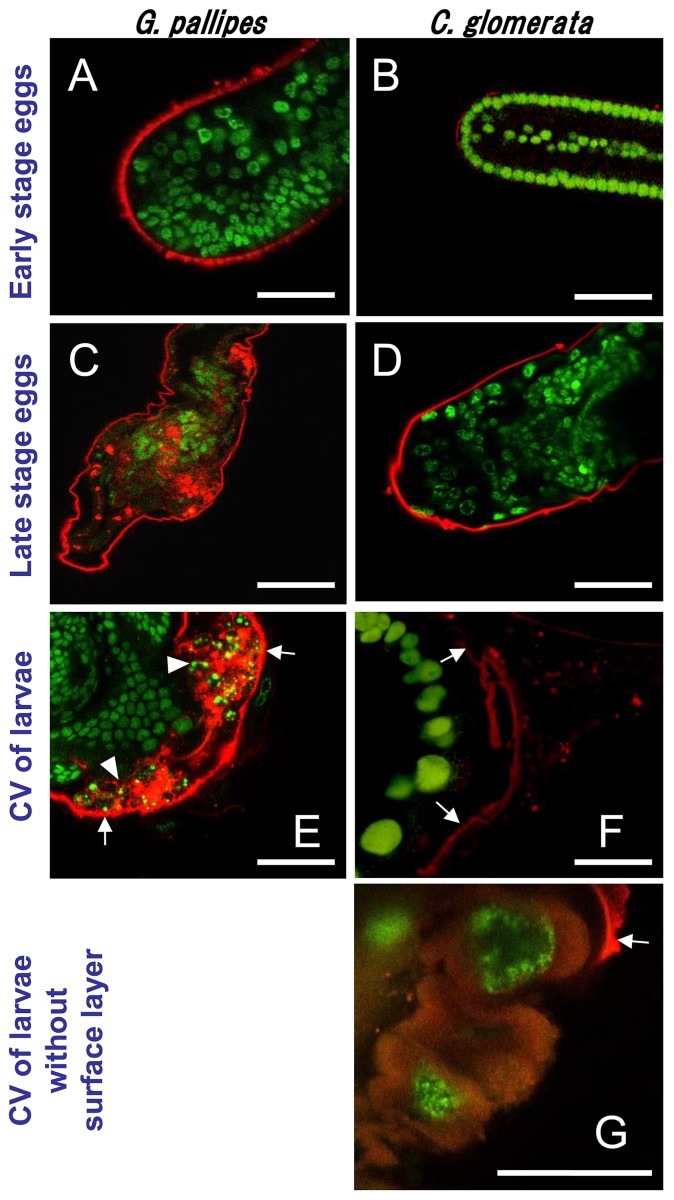
Penetration of pierisin-1 through parasitoid surfaces. A–F: Each stage of parasitoids was cultured with HiLyte dye-labeled pierisin-1 (red) for 24 h, and then labeled with STYO-16 Live-Cell Nucleic Acid Stain (green). CV, caudal vesicle. Arrows indicate surface layer of caudal vesicles. Apoptosis was observed in *G. pallipes* larvae (arrow heads). Parasitoid eggs and larvae were treated with Hilyte-labeled pierisin-1 at 10 µg/ml in the medium. The numbers of early stage eggs, late stage eggs, larvae and larvae without surface layer for *G. pallipes* and *C. glomerata* used for analysis were three to five. G: Incorporation of pierisin-1 at 100 µg/ml in the caudal vesicle cells of *C. glomerata* larvae after removal of the surface layer. The arrow indicates the surface layer of the caudal vesicle. The anterior is on the left. Horizontal bars represent 50 µm.

In order to clarify whether pierisin-1 penetrates parasitoid cells, permeability of pierisin-1 was examined using HiLyte dye-labeled pierisin-1 at 10 µg/ml. As shown in Figs. 3A and 3B, early stage eggs of *G. pallipes* and *C. glomerata* did not take up either pierisin-1 or BSA. In contrast, in the late stage eggs, penetration of HiLyte dye-labeled pierisin-1 could be seen in the non-habitual parasitoid *G. pallipes*, but not in the *C. glomerata*. Fig. 3C and d illustrate the incorporation status of pierisin-1 in late stage eggs of *G. pallipes* and *C. glomerata*, respectively. Similarly, in the case of second instar larvae, pierisin-1 was incorporated into caudal vesicle cells of non- permissive three wasps (Fig. 3E). However, in the case of *C. glomerata*, Hilyte dye-labeled pierisin-1 was observed on the exterior of the surface layer of the caudal vesicle, but not inside cells (Fig. 3F). On the other hand, incorporation of HiLyte dye-labeled BSA could be observed in late stage eggs and second instar larvae of all four wasps (data not shown).

As mentioned above, *C. glomerata* eggs and larvae were resistant to pierisin-1 toxicity, suggesting that the eggshell or surface layer of the larval caudal vesicle might be impervious to pierisin-1 incorporation. To examine whether inner cells in the larval caudal vesicle of *C. glomerata* are also resistant to pierisin-1, the surface layer of the caudal vesicle was removed, followed by incubation with Hilyte dye-labelled pierisin-1 at 100 µg/ml. As shown in Figure 3G, pierisin-1 penetrated into inner cells in the caudal vesicle of *C. glomerata*. Furthermore, many dead cells were observed in inner cells of *C. glomerata* treated with pierisin-1 (data not shown).

### Change of expression of pierisin-1 mRNA and protein after parasitization of larvae

**Figure 4:**
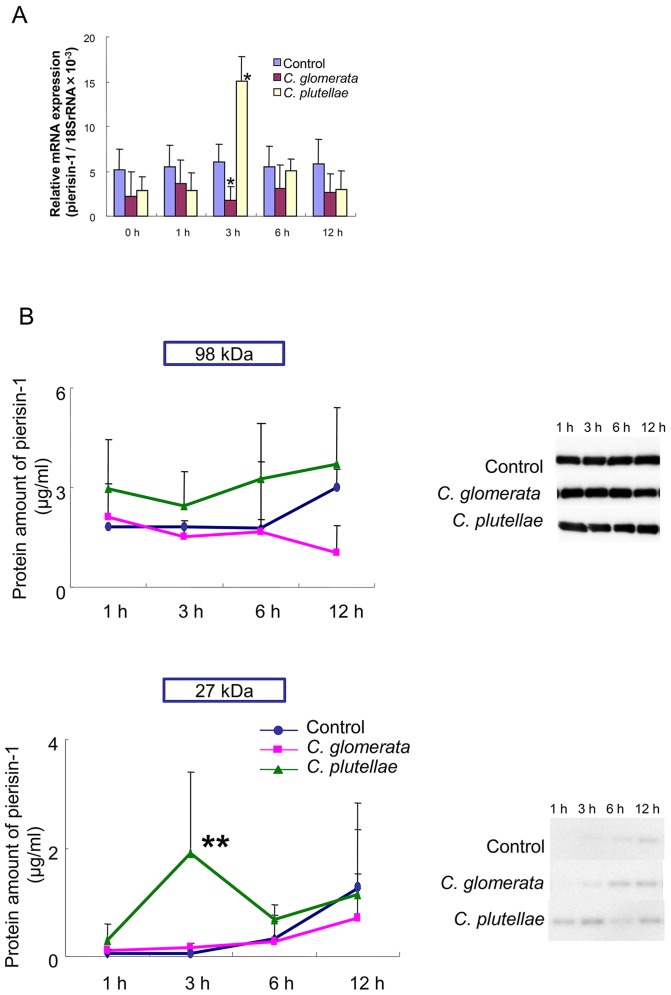
Alteration of pierisin-1 expression by parasitization. A: mRNA levels were analyzed by real-time PCR after parasitization. Blue column, control: red column, *C. glomerata*; yellow column, *C. plutellae*. The numbers of mRNA molecules per one thousand 18 S ribosomal RNA were calculated. Values are the means ± S.Ds. of three analyses. The numbers of samples were five each at each time point. * p<0.001, when compared to control. B: Western blotting analysis of pierisin-1 protein in the hemolymph 1–12 h after parasitization by *C. glomerata* or *C. plutellae*. Amounts of full-length pierisin-1 (98-kDa) and 27-kDa N-terminal fragments of pierisin-1 were separately calculated using an imaging system, as mentioned in Materials and Methods. Circles control: squares, *C. glomerata*; triangles, *C. plutellae*. The numbers of samples were five each at each time point.▪ p<0.001, when compared to controls.

The time course of pierisin-1 gene expression after parasitization was analyzed by real-time PCR using third-instar larva of *P. rapae.* We employed as parasitoids *C. plutellae* and *C. glomerata.* Both of them parasitize the hosts which eat same crucifer plants. Total RNA was extracted from the whole body at different time intervals after parasitization, and mRNA abundance was calculated as the number of mRNA molecules per 18S ribosomal RNA. As shown in Fig. 4A, pierisin-1 mRNA expression was increased around three - fold at 3 h after parasitization by *C. plutellae*, and then decreased to the normal level. In contrast, pierisin-1 expression tended to be decreased throughout the time course after parasitization by *C. glomerata*, and its level was significantly reduced at 3 h compared to the control level, suggesting that *C. glomerata* suppresses pierisin-1 expression.

The time course of expression of pierisin-1 protein after parasitization by *C. plutellae* and *C. glomerata* was also analyzed (Fig.4B). Protein amounts of pierisin-1 (98-kDa) at four time points (1, 3, 6 and 12 h) after treatment with *C. plutellae* tended to be higher than those with *C. glomerata* and control samples, although not statistically significant. On the other hand, protein amounts of pierisin-1 (98-kDa) with *C. glomerata* parasitization were similar to those with control samples at 1, 3 and 6 h, and slightly reduced at 12 h. On treatment of purified pierisin-1 with protease, pierisin-1 is reported to be nicked to generate 71-kDa C-terminal and 27-kDa N-terminal fragments, resulting in marked enhancement of ADP-ribosyltransferase activity[30]. As shown in Fig.4B, pierisin-1 proteins in hemolymph were cleaved in *P. rapae* parasityzed by *C. plutellae*, and the levels of 27-kDa N-terminal fragments, catalyzing ADP-ribosylation, were significantly increased at 3 h post parasitization. From these observations, it is suggested that parasitization by *C. plutellae* but not by *C.glomerata* enzymatically activates pierisin-1, in the larvae of *P.rapae*.

## Discussion

In the present study, we found that pierisin-1 caused cellular damage to eggs and larvae of non-habitual parasitoids for *P.rapae*, *C. plutellae*, *C. kariyai* and *G. pallipes* at doses of 1–100 µg/ml, essentially equivalent to those in first-instar to fifth-instar larvae of *P.rapae*. In contrast, pierisin-1 did not show any toxicity to eggs and larvae of the natural parasitoid of *P.rapae, C.glomerata*. Moreover, mRNA expression of pierisin-1 was increased at 3 hr after parasitization with *C.plutellae* in the larvae of *P.rapae*, but was decreased with *C.glomerata* parasitization. Activated 27 kDa pierisin-1 protein was also produced with *C. plutellae* parasitization. Thus, pierisin-1 may play some role as a defense factor against parasitization by some types of wasps in *P.rapae.*

Pierisin-1 caused damage to cells in the late stage parasitoid eggs and the caudal vesicle of parasiatoid larvae of *C. plutellae*, *C. kariyai* and *G. pallipes*. It has been reported that late stage parasitoid eggs absorb nutrients from host haemolymph through egg shells [31], [32]. In addition, it is thought that the caudal vesicle of parasitoid larvae absorbs nutrients and oxygen from host hemolymph [32], [33]. In fact, we found that late stage eggs and the caudal vesicle of parasitoid larvae absorbed BSA. Because the shells of late stage eggs and the caudal vesicle are known to be permeable structures, hemolymph components like pierisin-1 could be permitted to go through and then cause the cellular damage in the three non-habitual parasitoids. On the other hand, the dominant gregarious endoparasitoid of *P. rapae*, *C. glomerata* eggs and larvae were resistant to pierisin-1 toxicity. Moreover, we found that pierisin-1 penetrated into inner cells in the caudal vesicle of *C.glomerata* to induce cell death when the surface layer was removed. Thus, it is possible that *C.glomerata* has acquired tolerance to the toxicity of pierisin-1 by inhibiting its penetration through egg shells and the surface layers of larvae during evolution.

Humoral defense factors such as phenoloxidase cascade for melanization [12], [13], [34] and antimicrobial peptides [10], [11] are activated when foreign objects invade. Although pierisin-1 protein is a resident component in hemolymph, pierisin-1 expression was increased and additionally activated when non-habitual parasitic wasp, *C. plutellae*, parasitized *P.rapae.* These results strongly suggest that pierisin-1 is involved in the elimination system against parasitic wasps. Normally, pierisin-1 is mainly expressed in fat bodies [9]. Putative transcription factors of DNA-binding motifs for expression of each anti-microbial peptide are located in the -1000 base pair upstream regions of the pierisin-1 gene[35]. It is reported that parasitism induces relatively few changes in expression of antimicrobial effector genes under Toll and imd pathway control [36], [37], [38]. Pierisin-1 induction may be regulated by these important overlapping genes. In the white cabbage butterfly following pattern-recognition, piersin-1 is likely to be induced by both Toll pathway responding to latex beads or Gram positive bacteria, and imd pathway responding to non-habital parasitoid wasps including *C. plutellae, C. kariyai* and *G. pallipes* or Gram negative bacteria. Our preliminary result indicate that the non-habitual parasitic wasp *C. plutellae* was shown to induce pierisin-1 expression mainly in fat bodies and hemocytes of *P. rapae*. These foreign species probably have both ligands for recognition receptors for the Toll and imd pathways. Since the regulation factors of pierisin-1 expression might span multiple pathways, this also supports the conclusion that pierisin-1 has important roles in the innate immune system of cabbage white butterfly.

On the other hand, parasitization by the natural parasitoid, *C. glomerata*, suppressed pierisin-1 expression. It is well known that natural parasites, including parasitic wasps and malaria parasites, suppress harmful host immune systems such as melanization, hemocyte spreading and encapsulation [39], [40] [17], [39], [40]. In Cotesia parasitoids as well, the egg surface fibroblast layer, maternal venom and polydnavirus existing in the calyx fluid of maternal oviducts acts in evasion of host defenses [41], [42], [43], [44], [45], and suppress the hemocyte functions contributing to encapsulation [46], [47], [48], [49], [50], [51], [52]. By similar mechanisms, expression of pierisin-1 may also be suppressed by parasitization of *C. glomerata* in *P.rapae*.

Trypsin-treated pierisin-1 which is considered to be a ‘nicked’ full-length form composed of associated N- and C-terminal fragments[30] demonstrates strong cytotoxic and apoptosis-inducing activities, five-fold greater against mammalian cancer cells and one hundred times elevated against *G. pallipes* larvae (data not shown). The humoral immune response involves proteolytic cascades leading to melanization via a prophenoloxidase cascade[53], [54]. The Toll pathway and melanization reactions share a common serine protease for the regulation of these two major innate immune responses [55], [56]. Our preliminary experimental data indicate that pierisin-1 protein in hemolymph of *P. rapae* is cleaved by the action of hemocytes mainly in the presence of non-habitual wasp eggs. Serine proteases, which play some important roles in innate immune responses, may also contribute to activate pierisin-1 protein in *P. rapae*.

In addition to pierisin-1, three kinds of pierisin-1-like proteins have been identified; pierisin-2 from *P. brassicae*, pierisin-3 from *P. melete* and pierisin-4 from *Aporia crataegi* [*6*], [*7*]. Pierisin-2, −3 and −4 show similar properties to pierisin-1, and these proteins are also considered to play some role in the developmental stages because the larvae and the pupae of *P.brassicae, P.melete* and *A.crataegi* exhibit higher cytotoxicity than the adults [5]. It is also possible that pierisin-2, −3, and −4 protect against invading organisms, such as parasitic wasps, as in the case of pierisin-1. The biological significance of pierisin-2,−3 and −4 in Pierina butterflies is now under investigation.

Like Sarcophaga lectin and sapecin [57], pierisin-1 could be involved in both defense systems and metamorphosis in *P. rapae*. Existence of non-habitual wasp eggs could be recognized by hemocytes or recognition components of plasma, then encapsulation by hemocytes begins. This information is transmitted to fat body cells and pierisin-1 mRNA is induced. At the same time, hemocytes probably secrete serine proteases and then pierisin-1 protein in plasma is activated regionally to kill parasitic wasps efficiently. Further analyses may reveal more detailed roles of DNA ADP-ribosylating proteins in insect immune systems.
